# Draft Genome Sequences of Two Lipopeptide-Producing Strains of *Bacillus methylotrophicus*

**DOI:** 10.1128/genomeA.01176-15

**Published:** 2015-10-08

**Authors:** Julie Jeukens, Irena Kukavica-Ibrulj, Luca Freschi, Suha Jabaji, Roger C. Levesque

**Affiliations:** a Institut de Biologie Intégrative et des Systèmes (IBIS), Université Laval, Québec, Québec, Canada; b Plant Science Department, Faculty of Agricultural and Environmental Sciences, MacDonald Campus, McGill University, Montreal, Québec, Canada

## Abstract

*Bacillus methylotrophicus* is implicated in phytostimulation and disease suppression of agricultural and bioenergy crops. Here, we present the genome sequences of *B. methylotrophicus* strains B26 and OB9. Their assembly resulted in 26 and 24 contigs, respectively. These strains are well suited for comparative genomics studies and the evaluation of commercially valuable biomolecular compounds.

## GENOME ANNOUNCEMENT

*Bacillus methylotrophicus* is a recently described Gram-positive bacterium (1), which, like other species of the genus, is associated with plant roots and exerts beneficial effects on plant development (2–5). It competitively colonizes plant roots, may internalize plant tissues, and can simultaneously act as biofertilizer and antagonist (biopesticide) of recognized root pathogens, including Gram-negative bacteria and fungi (4, 6). Phytostimulation and disease suppression are partly linked to the production of secondary metabolites and antibiotics (5). This species is similar to *Bacillus subtilis* and is considered a successful biological control agent due to an endospore-forming lifestyle that facilitates the development of commercial products with long-term viability (7). Strain B26, previously described as *B. subtilis* B26 and isolated from the bioenergy crop switchgrass (4), and strain OB9, from crude oil, both produce lipopeptides, such as surfactins. These biomolecules, in addition to their antimicrobial activity (8), act as immunostimulators of the host plant (5) and have gained importance in the fields of environmental bioremediation, food processing, and pharmaceuticals (9).

Genomic DNA was isolated from overnight cultures using the DNeasy blood and tissue kit (Qiagen, Hilden, Germany) and according to manufacturer's instructions for Gram-positive bacteria. Genomic DNA (500 ng) was mechanically fragmented for 40 s using a Covaris M220 (Covaris, Woburn, MA, USA) with the default settings. Fragmented DNA was transferred to PCR tubes, and library synthesis was performed with the Kapa Hyper Prep kit (Kapa Biosystems, Wilmington, MA, USA), according to the manufacturer’s instructions. TruSeq HT adapters (Illumina, San Diego, CA, USA) were used to bar code the libraries, which were each sequenced in 1/48 of an Illumina MiSeq 300-bp paired-end run at the Plateforme d’ Analyses Génomiques of the Institut de Biologie Intégrative et des Systèmes (Laval University, Quebec, Canada). Sequencing datasets were assembled *de novo* with the A5 pipeline (10).

The B26 genome assembly consists of 26 contigs (median coverage, 85×), for an estimated total size of 3,868,758 bp, while the OB9 genome assembly consists of 24 contigs (median coverage, 54×), for an estimated total size of 3,861,454 bp. Core genome phylogeny with the Harvest suite (11) and whole-genome BLASTn searches (12) revealed that the genomes of strains B26 and OB9 are closely related to one another and are part of the same clade as *Bacillus amyloliquefaciens* subs. *plantarum* isolate UCMB5113 (13). However, it was recently demonstrated that this taxon is paraphyletic and should be included in *B. methylotrophicus* (14).

These strains are well suited for comparative genomics with other *Bacillus* strains and the evaluation of new biomolecular compounds that are valuable for commercial production.

### Nucleotide sequence accession numbers.

These whole-genome shotgun projects have been deposited at DDBJ/EMBL/GenBank under accession numbers LGAT00000000 (B26) and LGAU00000000 (OB9).
